# Perinatal outcomes of women with hypertensive disorders of pregnancy in Jimma Medical Center, southwest Ethiopia: Retrospective cohort study

**DOI:** 10.1371/journal.pone.0256520

**Published:** 2021-08-19

**Authors:** Debela Dereje Jaleta, Tadesse Gebremedhin, Mulusew Gerbaba Jebena

**Affiliations:** 1 Department of Nursing, College of Health Sciences, Mettu University, Mettu, Ethiopia; 2 Faculty of Public Health, Department of Epidemiology, College of Health Sciences, Jimma University, Jimma, Ethiopia; Universita degli Studi dell’Insubria, ITALY

## Abstract

**Background:**

Hypertensive disorders of pregnancy (HDP) increases adverse perinatal outcomes in women with the disorder. About 16% of all still births and 10% of early neonatal deaths are accounted by HDP. In Ethiopia, HDP complicates about 6% of all pregnancies. Hence, the objective of this study was to determine the risk of adverse perinatal outcomes among women with HDP in Jimma Medical Center, southwest Ethiopia.

**Methods:**

A retrospective cohort study was conducted on a total of 777 women who gave birth from June 2017 to March 2020 at Jimma Medical Center, southwest Ethiopia. Women with HDP and normotensive women who gave birth at or after 28 weeks of gestation were enrolled as exposed and unexposed respectively. Simple random sampling technique was used to select study participants. Data were reviewed using structured data collection performa that was prepared after reviewing relevant literatures. Data were entered to Epi-Data then exported to STATA version 13 for analysis. The adverse perinatal outcomes risk were examined using log binomial and modified Poisson regression model with robust standard errors.

**Results:**

In this study, the overall incidence of adverse perinatal outcome was higher among women with hypertensive disorders of pregnancy (HDP) than normotensive women (64.1% versus 32.8%). After adjusting for confounders, women with HDP were at higher risk of babies with low birth weight (adjusted RR = 2.88 (2.2, 3.75)), preterm birth(aRR = 2.31(1.7, 3.14)), fifth minute low Apgar score (aRR = 2.6(1.53, 4.42)), admission to neonatal intensive care unit (aRR = 1.77(1.32, 2.37), stillbirth (aRR = 2.02(1.11, 3.01)), and perinatal mortality (aRR = 3.88(1.97, 7.66)) than normotensive women.

**Conclusion:**

Women with hypertensive disorder of pregnancy were at higher risk of adverse perinatal outcomes than normotensive women who gave birth at Jimma Medical Center, southwest Ethiopia. Therefore, health care providers should strengthen prevention, early diagnosis and prompt management of HDP in order to reduce adverse perinatal outcomes.

## Introduction

Hypertensive disorders of pregnancy (HDP) are multisystem diseases, which include chronic hypertension (preexisting), gestational hypertension, preeclampsia, eclampsia, and superimposed preeclampsia on chronic hypertension [1]. Hypertensive disorders affects 10% of pregnancies around the globe and it is an important cause of maternal and perinatal morbidity [1–3].However, it is disproportionally distributed across the world, of that estimated magnitude of HPD in developing countries reaches up to 16.7% [4].

In addition to that, studies conducted in Ethiopia revealed a high burden of HDP which ranges from 2.23 to 18.25% [5–7]. Similarly, the study conducted in Jimma University specialized hospital reported 8.5% of hypertensive disorders of pregnancy, which is higher than the national pooled prevalence of 6.29% [6,8].

HDP is associated with disturbed vascular manifestations, oxidative stress and endothelial damage. This affects placental function resulting in poorer perfusion and nutrient supplementation to the fetus that enhance adverse perinatal outcomes [9].

HDP accounts for 15% of perinatal deaths globally [10,11]. Among the estimated 2.6 million stillbirths annually, approximately 16% occur in pregnancies complicated by hypertension disorder of pregnancy [12]. It has been estimated that the HDPs precede 10% of early neonatal deaths (8/1000 live births) [13]. Multi-country study conducted by WHO, eclampsia/pre-eclampsia was the primary obstetrical cause accounting for 1 out of 4 perinatal deaths, with similar proportions affected by stillbirths and early neonatal deaths [14]. The study conducted in 29 low and middle-income countries, reported that eclampsia and pre-eclampsia underlying 7.5% of macerated late fetal deaths, 9% of fresh late fetal deaths, and 10% of early neonatal deaths [15].

Hypertensive disorders of pregnancy account about 7% of perinatal mortality causes in Ethiopia which may be responsible for highest perinatal mortality rate in Sub Saharan Africa [9]. Prevalence of perinatal and maternal mortality among pregnant women with hypertensive disorders was found to be higher than rates reported from high income [6]. One in four pregnancies complicated by hypertensive disorder end up in perinatal death and newborn with low birth weight was found to be 37% [7]. The 5-minute APGAR score of <7 in women with HDP was highest (52.8%) in Addis Ababa [16] and the lowest rate being 13.4% in Southern Ethiopia [17]. Preterm birth complicates as high as 65.3% of women with HDP in Somalia regional state Ethiopia [18] to as low as 31% in Oromia regional state [19].

Even though, hypertensive disorders of pregnancy have adverse outcomes on both maternal and newborns; the risk of maternal death is less than 1% in severe preeclampsia, whereas perinatal death was about 13%. The situation is worse in eclampsia; where the risks of maternal and perinatal deaths occur in about 5% and 28% respectively [20]. Study from India reported the rate of maternal mortality was 5.55% and perinatal deaths occurred in 37.5% of the deliveries [21].

Despite the fact that, most obstetricians worry more about the risk of maternal death in women whose pregnancies are complicated by hypertensive disorders, but the risk of perinatal outcome is more overwhelming [20]. HDP is associated with higher rates of morbidity and mortality including intrauterine growth restriction, intrauterine fetal death, preterm birth, birth asphyxia, low birth weight and perinatal mortality [22–25].This study aimed to show, adverse effect of hypertensive disorders of pregnancy on a number of important perinatal outcomes in women with HDP compared to normotensive pregnant women gave birth at JMC in order to improve maternal and child health care.

## Methods and materials

### Study setting, design and participants

This study was conducted in Jimma Medical Center (JMC) which is located in Jimma town, southwest Ethiopia, 343km away from Addis Ababa, the capital city of Ethiopia. Approximately, more than 15 million people are served by Jimma Medical Center. During study time period 9,590 deliveries were conducted at JMC. The total numbers of children in the JMC catchment areas are 2,250,000 and it has 1600 staff members, 32 intensive care units, and 800 beds. A retrospective cohort study was conducted using document review of women with hypertensive disorder of pregnancy and normotensive women who gave birth at Jimma Medical Center from June 26, 2017- March 16, 2020. The source population for this study was all pregnant mothers with hypertensive disorder of pregnancy as the exposed group and all normotensive mothers as the non-exposed group, who gave birth during the study period. HDP encompasses chronic hypertension, gestational hypertension, preeclampsia/eclampsia, and superimposed hypertension. Hypertension in pregnancy is defined as systolic blood pressure greater than or equal to 140 mmHg and/or diastolic blood pressure greater than or equal to 90 mmHg which usually confirmed within four hours apart measurement and normotensive women as of systolic blood pressure ≤140 mmHg and diastolic blood pressure ≤90mmHg. Women with missing record of gestational age at birth, age of newborn at death, birth weight, and multiple births more than twin and birth with congenital anomalies in child perspective and mothers with DM, heart disease, incompetent cervix and Rh sensitization were excluded. Sample size was calculated by using STATCALC command of Epi-info version 7 with the following assumption of 95% confidence level, 80% power, ratio of unexposed to exposed of 2, by taking 1.7% of still birth among healthy mothers (normotensive) to detect the minimum relative risk of 3.46 [26]. Accordingly the sample size was approximately **777** with 259 women diagnosed with HDP and 518 normotensive women. Maternity ward health management information system (HMIS) log book was used to obtain as an entry point to identify women with their exposure status using their medical record number. Sampling frames were prepared and simple random sampling technique without replacement method was employed to select individual medical record separately for both exposed and non-exposed women using computer random number generator.

### Variables

**Dependent:** Perinatal outcomes (preterm birth, low birth weight, small for gestational age, first and fifth minute APGAR score of <7, still birth, admission to NICU, early neonatal death, perinatal mortality). **Exposure:** Blood pressure of sustained systolic BP ≥140 mmHg or diastolic BP ≥ 90 mmHg based on the average of at least two measurements, using the same arm for measurement.

Adverse perinatal outcome is defined as a newborn with the occurrence of any of the following outcomes: low birth weight, low Apgar score, small for gestational age, preterm delivery, admission to neonatal intensive care unit and perinatal death. Low birth weight was defined as a birth weight of alive born infant of less than 2,500 g. Preterm delivery is a birth of infants occurring after 28 completed weeks but before 37 completed weeks of gestation. Small for gestational age is defined as a birth weight of newborn below the tenth percentile of weight distribution at the specified gestational age of a pregnancy. A low Apgar score, when 1^st^ minute and 5th minute Apgar score is less than 7. Stillbirth is death prior to the complete expulsion or extraction from its mother of a product of conception after 28 weeks of pregnancy; the death is indicated by the fact that after such separation the fetus does not breathe or show any other evidence of life, such as beating of the heart, pulsation of the umbilical cord or definite movement of voluntary muscles. Early neonatal deaths are a death that occurred in the first week of neonatal life before discharge from the study hospital after 28 weeks of gestation. Perinatal mortality is stillbirths and newborn deaths within the first seven days of delivery [26–30].

### Data collection tools and procedure

Data were collected from the medical record by using structured data collection format that was prepared after reviewing literatures [26–30]. Six health professionals were recruited to collect data from maternal and neonatal records. Two day training was conducted and they were explained about the objective of the study, and trained how to fill the performa by taking 5% of samples of cards from archive of JMC. To ensure the completeness of information during data collection, newborn information such as sepsis diagnosis, early neonatal status were extracted from neonatal intensive care unit logbook by admission date, sex, mother’s name and age at death.

### Data processing and analysis

Data were entered into Epi data manager version 3.1 and exported to STATA version 13 software package for analysis. Descriptive statistics (frequency and percentage for categorical variables and summary statistics for continuous variables were used to characterize the study population and the median difference between groups for continuous variables such as; age of the mother, gestational age and birth weight were tested using Mann-Whitney test because of failed normality assumption. Bivariate regression was done to select independent variables for all outcome variables using p-value cut of point≤0.25 to be considered as candidate variables for multivariable regression analysis. The relative risk of adverse perinatal outcomes (small for gestational age and early neonatal death) were calculated using log-binomial model. As a first approach to the multivariable analysis, log-binomial models were used for all outcome variables. But, owning to the sparseness of data, failed for convergence assumption encountered for still birth, low birth weight, first and fifth minute low APGAR scores, preterm birth, Admission to NICU and perinatal mortality. Therefore, modified Poisson regression were opted with lowest Akaike’s information criteria (AIC) and Bayesian information criteria (BIC) to select the final model using backward methods. For early neonatal death and small for gestational age, log-binomial models were used. Maternal age, residence place, marital status, sex of the newborn, gravidity, mode of delivery, history of anemia, fetal mal-presentation at birth, ANC follow up, malaria infection, ante-partum hemorrhage, venereal disease research laboratory (VDRL) test, platelet count, onset of labour, maternal outcome and maternal HIV status were controlled in the statistical models. After adjusted for confounders relative risk with 95% confidence interval and p-value < 0.05 was considered to declare statistical significance.

### Ethical consideration

This research was conducted after receiving ethical clearance letter from Jimma University, institute of health science, institutional review board Ref No IRB/00099/2020; date of issue 20/03/2020. Medical records of women who gave birth from June 26, 2017 to March 16, 2020 at Jimma Medical Center were reviewed. All methods were performed following principles of Helsinki declaration. Jimma University institute of health science review board waived off the need for participant informed consent during ethical review for the fact that, it was not possible to reach individuals to whom the reviewed charts belongs to, rather formal letter of cooperation was written to Jimma medical center administration from Jimma University. Written permission was secured from the Jimma Medical center, medical director office.

## Results

### Socio-demographic and obstetric characteristics of study participants

Overall, a total of 259 women with hypertensive disorders of pregnancy and 518 normotensive pregnant women documents were reviewed in the study. Among a total of 259 women with HDP’s participated in the study: 3.9%, 10.4%, 65.6% and 20.1% had chronic hypertension, gestational hypertension, pre-eclampsia and eclampsia respectively.

The median age of the mothers with HDP and normotensive women’s were 26(inter-quartile range (IQR) = 8) and 27(IQR = 6) years respectively and there was no significant age difference between both group using Mann-Whitney U test of p = 0.104. Moreover, 136 (52.5%) of mothers with HDP and 211 (40.7%) of the controls were rural dwellers. The proportion of primigravidas was higher among women with HDP than normotensive women (41.7% versus 33%).The proportion of having anemia was roughly similar among women with HDP and normotensive women (21.6% versus 19.1%). The number of ANC visit among mothers with HDP and normotensive women were statistically different with X^2^ = 9.35, p = 0.009. From the total deliveries attended, 61.8% of women with HDP and 42.1% of normotensive women gave birth vaginally from each group [***Table 1***].

**Table 1:** Socio-demographic and obstetric characteristics of pregnant women who gave birth at JMC in Jimma town, southwest Ethiopia from June, 2017- March, 2020.

Variable		Women with HDP Frequency (%)	Normotensive women Frequency (%)
Residence	Urban	123 (47.5)	307 (59.3)
	Rural	136 (52.5)	211 (40.7)
Marital status	Single	3(1.2)	3 (0.6)
	Married	256 (98.8)	515 (99.4)
Gravidity	Primigravida	108 (41.7)	171 (33)
	Multipara (II–IV)	110 (42.5)	272 (52.5)
	Grand multipara (V+)	41 (15.8)	75 (14.5)
History of anemia	Yes	56 (21.6)	99 (19.1)
	No	203 (78.4)	419 (80.9)
Malaria diagnosed current pregnancy	Yes	21 (8.1)	20 (3.9)
	No	238 (91.9)	498 (96.1)
Maternal HIV status	Reactive	11 (4.25)	12 (2.3)
	Non-reactive	248 (95.8)	506 (97.7)
VDRL test positive	Yes	4 (1.5)	5 (1.0)
	No	255 (98.5)	513 (99)
Number of antenatal care visit in current pregnancy	Zero	11 (4.3)	12 (2.3)
	1 to 4	208 (80.3)	381 (73.6)
	5+	40 (15.4)	125 (24.1)
On set of labour	Induced	101(39)	48 (9.3)
	Spontaneous	122 (47.1)	385 (74.3)
	Direct cesarean Section	36 (13.9)	85 (16.4)
Fetal mal-presentation at birth	Yes	31 (12)	129 (24.9)
	No	228 (88)	389 (75.1)
Platelet count	100,000+	225 (86.9)	511 (98.6)
	<100,000	34 (13.1)	7 (1.4)
Mode of delivery	Vaginal	160 (61.8)	218 (42.1)
	Cesarean Section	99 (38.2)	300 (57.9)
Type of pregnancy	Single	249 (96.1)	498 (96.1)
	Twin	10 (3.9)	20 (3.9)
Maternal outcome	Alive on discharge	253 (97.7)	517 (99.8)
	Died	6 (2.3)	1 (0.2)
Maternal Age (year)		**Median (IQR)**	**Median (IQR)**
		26 (IQR = 8)	27(IQR = 6)

### Incidence of adverse perinatal outcomes among study participants

About 64.1% of women with HDP and 32.8% of normotensive women developed adverse perinatal outcomes The incidence of still birth was higher among newborn babies delivered from women with HDP than normotensive women (11.2% versus 4.1%).The stillbirth rate among women with HDP was 112/1000 total births. Similarly, first and fifth minute low APGAR scores of the babies were higher among women with HDP than normotensive women (37.8% versus 19.5%), (12.6% versus 4.4%) respectively. Prematurity among mothers with HDP and normotensive mothers was about 39.4% and 10.6% respectively [***Table 2***]. Women with HDP were more likely to have a lower gestational age at delivery than normotensive women with median gestational age of (37 with IQR = 3 weeks versus 39 with IQR = 3) weeks, p < 0.001. Low birth weight babies of mothers with HDP and normotensive were39.8% and 12.7% respectively. Women with HDP were more likely to have a low birth weight babies than normotensive women at p < 0.001. Median birth weight and IQR of babies born to women with HDP and normotensive were 2600g (IQR = 1000g) and 3200g (IQR = 740g) respectively.

**Table 2:** Incidence of adverse perinatal outcomes with confidence interval among women with HDP and Normotensive women who gave birth at JMC, June, 2017 to March 2020.

Perinatal outcomes	Incidence of adverse perinatal outcome with (CI) among Women with HDP(n = 259)	Incidence of adverse perinatal outcome with (CI) among Normotensive women (n = 518)
Stillbirth	11.2 (7.63–15.68)%	4.1 (2.53–6.13)%
First low minute APGAR score*	37.8 (31.53–44.44)%	19.5 (16.12–23.28)%
Fifth minute low APGAR score*	12.6 (8.61–17.6)%	4.4 (2.79–6.63)%
Small for gestational age(SGA)	9.3 (6.03–13.47)%	2.3 (1.2–4.01)%
Low birth weight	39.8 (33.76–46.01)%	12.7(9.99–15.92)%
Preterm birth	39.4 (33.39–45.61)%	10.6(8.1–13.6)%
NICU admission*	33.9 (27.82–40.43)%	15.1(12.06–18.54)%
Early neonatal death (ENND)*	11.3 (7.52–16.12)%	2.2 (1.11–3.93)%
Perinatal mortality(PNM)	21.2 (16.42–26.73)%	6.2 (4.26–8.61)%
Overall adverse perinatal outcome	64.1 (57.9–69.9)%	32.8 (28.78–37.05)%

For all with the * (n = 230 for women with HDP and n = 497 for normotensive women).

Overall perinatal death in women with HDP was 21.2% and 6.2% among normotensive women. Similarly, the perinatal mortality rate among women with HDP was 212 per 1000 total births and 62 per 1000 total births among normotensive women. Incidences of all adverse perinatal outcomes were higher among women with HDP. Incidence of all adverse perinatal outcomes between women with HDP and normotensive women has statistical significance difference between both groups [***Table 2***].

### Risk of adverse perinatal outcomes associated with hypertensive disorders of pregnancy

Risk estimates for adverse perinatal outcomes among women with HDP compared with normotensive women are shown in **[*Table 3*]**. In the final multivariable analysis, women with HDP were significantly at higher risk of: preterm birth with adjusted RR: 2.31at 95%CI of (1.7, 3.14)), still birth; adjusted RR:2.02 at 95%CI of (1.21,3.40), first minute low APGAR score; adjusted RR: 1.93 at 95%CI of (1.52, 2.46), fifth minute low APGAR score adjusted RR: 2.6 at 95% CI of (1.53, 4.42), small for gestational age; adjusted RR: 3.21at 95% CI of (1.56, 6.58), admission to NICU adjusted RR: 1.77 at 95% CI of (1.32, 2.37), low birth weight; adjusted RR: 2.88 at 95% CI of (2.2, 3.75), and perinatal mortality; adjusted RR: 3.88 (1.97, 7.66) were significantly higher in women with hypertensive disorders of pregnancy compared to normotensive women after adjusted for maternal age, residence place, marital status, sex of the newborn, gravidity, mode of delivery, history of anemia, fetal mal-presentation at birth, ANC follow up, malaria infection, ante-partum hemorrhage, VDRL test, platelet count, onset of labour, maternal outcome and maternal HIV status.

**Table 3:** Bivariate and multivariate analysis on the association between HDP and adverse perinatal outcomes among women gave birth from June, 2017 to March, 2020 at JMC, southwest Ethiopia.

Outcomes Variable		Women with HDP(n = 259)	Normotensive women(n = 518)	Unadjusted RR (95% CI)	Adjusted RR (95% CI)
Stillbirth**	Yes	29	21	2.76(1.61, 4.75)	2.02 (1.11, 3.01)
	No	230	497	1	1
First minute low APGAR score **	Yes	87	97	1.94(1.51, 2.47)	1.93 (1.52, 2.46)
	No	143	400	1	1
Fifth minute low APGAR score**	Yes	29	22	2.85(1.67, 4.85)	2.6 (1.53, 4.42)
	No	201	475	1	1
Small for gestational age(SGA)*	Yes	24)	12	4(2.03, 7.87)	3.21(1.56, 6.58)
	No	235	506	1	1
Low birth weight*	Yes	103	66	3.12(2.38, 4.09)	2.88 (2.2, 3.75)
	No	156	452	1	1
Preterm birth*	Yes	102	55	3.71(2.77, 4.97)	2.31 (1.7, 3.14)
	No	157	463	1	1
NICU admission**	Yes	78	75	2.25(1.71, 2.96)	1.77 (1.32, 2.37)
	No	152	422	1	1
Early neonatal death (ENND)**	Yes	26	11	5.11(2.57,10.16)	3.7(1.89, 7.15)
	No	204	486	1	1
Perinatal mortality(PNM)**	Yes	55	32	3.61(2.39, 5.46)	3.88 (1.97, 7.66)
	No	204	486	1	1

*RR- Relative Risk, were adjusted for maternal age, residence place, marital status, sex of the newborn, gravidity, history of anemia, ANC follow up, malaria infection, ante-partum hemorrhage, VDRL test, platelet count, maternal outcome and maternal HIV status.

**RR- relative risk were adjusted for all variable with * and additional for mode of delivery, fetal mal-presentation at birth, onset of labour and 1 represent control group.

## Discussion

This study aimed to determine adverse perinatal outcomes (preterm birth, first and fifth minute low Apgar score, stillbirth, small for gestational age, low birth weight, admission to NICU, early neonatal death, and perinatal mortality) associated with HDP among women who gave birth at Jimma Medical Center from June 2017 –March 2020. The findings of this study demonstrate that hypertensive disorders of pregnancy independently conferred an increased risk for adverse perinatal outcomes compared to normotensive pregnant women.

The finding of this study showed that 39.4%, 95% of CI: (33.39–45.61) of women with HDP gave preterm babies. This finding was similar to the study conducted in part of Ethiopia, Tigray region (40.8%), and Nekemte (41.2%) [26,31].In contrast, it was higher than a study conducted in Ghana (21.7%), India (24.6%), the US (17.4%), and São Paulo city (10.6%) [26,32–34]. The similarity in the incidence of preterm birth across the studies might be due to the similar quality of antenatal care service and the same guideline used for the management of HDP in the areas. The difference might be due to the level of ANC services quality and different management guidelines used across the countries.

Consequentially, there was a higher risk of delivering preterm babies among women with HDP than normotensive women, which was consistent with a study conducted in Nigeria, Tigray region, and US [26,35,36]. This could be due to interventional delivery being carried out irrespective of gestational weeks. Particularly, in women with severe preeclampsia and eclampsia subtype of HDP; early delivery carried out in order to prevent further maternal and perinatal adverse effect. However, prematurity is the leading cause of child deaths, accounting for nearly for18 deaths per 1000 live birth worldwide [37]. Thus, preventing and managing hypertensive disorders of pregnancy should become the priority to accelerate the progress for neonatal survival.

This study revealed that the incidence of low birth weight among women with hypertensive disorders of pregnancy was 39.8% and although, there was a statistically significant difference between the mean birth weight of mothers with HDP and normotensive mothers. This finding was similar with a study conducted in Nekemte (36.2%), Tigray region which reported (37.7%); Ethiopia [26,31] and higher than the studies conducted in Ghana (24.7%), Zimbabwe (16%), India (22.2%) and São Paulo city (21%) [26,32–34,36].

Incidence of low birth weight difference across the studies might be due to antenatal care service quality and management for HDP difference between the study areas. It is consistent with studies conducted in Nigeria and Tigray region [26,30]. This could be due to the HDP effect on vascular manifestation disturbance that affects placental function, which ends up poorer perfusion and nutrient supplementation to the fetus. Thus, reducing the risk of low birth weight demands; increased attention to keeping the newborn warm, including skin to skincare, and assistance with early initiation of breastfeeding.

The incidences of first and fifth minute low Apgar score among babies of women with HDP were 37.8% with 95% of CI: (31.5–44.4) and 12.6%, 95% of CI: (8.61–17.6) respectively. These findings were similar with a study conducted in Tigray region (40.8% for the first minute), Nigeria (11.9% for the fifth minute) and higher compared to studies conducted in Nigeria for the first minute (6.7%), Zimbabwe (8.9% for the first minute, 10% for the fifth minute) [26,30,36]. This discrepancy might be due to differences in study design and sample size, and improved early identification of high-risk mothers.

In addition, the risk of both first and fifth minute low Apgar score among newborn babies of women with HDP was higher; these were consistent with studies conducted in Ethiopia, Zimbabwe, and Nigeria [26,30,36]. This could be related to hypertensive disorders of pregnancy effect on vascular disturbance, oxidative stress, endothelial damage and increased preterm birth that might be vulnerable to the immaturity of muscle tone and reflex irritability. The lungs of preterm birth may be deficient in surfactant that makes the lung more difficult to ventilate. For the reason, all necessary equipment for newborn resuscitation should be ready at every delivery by anticipating the risk of birth with a low Apgar score among women with HDP, since newborn that does not start breathing on their own by 1 min after birth should receive positive pressure ventilation with room air by a self-inflating bag and mask.

Additionally, the incidence of newborn babies with small for gestational age among women with HDP was 9.3% with 95% of CI: (6.03–13.47). This was slightly similar with the study conducted in Ghana (6.3%) and lower than the study conducted in Nigeria (15.3%), South Africa (17%), Madagascar (25.7%) and Ethiopia; Tigray region (36.7%) [4,26,30,32,38]. This difference might be due to the frequency of antenatal care services utilization by pregnant women may vary across the areas. For instance, the proportion of antenatal care service visits in this particular study four times and more was 54.2%, which was higher than other study reports [39].

Moreover, the risk of small for gestational age among women with HDP was higher than normotensive women. This finding was consistent with studies conducted in Ethiopia and Nigeria [26,30]. This could be related to intrauterine growth restriction due to insufficient uteroplacental blood flow and the development of ischemia in women with hypertensive disorder of pregnancy.

According to this study, almost one third (33.9%), 95% of CI: (27.82–40.43) of the newborns delivered from women with HDP were admitted to the neonatal intensive care unit. This finding was roughly similar with others study reported from Ethiopia; Tigray region (28.8%), Nekemte (29.1%), and it was higher than studies reported from Iran (13%), Nigeria (14.7%), Egypt (18.8%) and India (25.5%) [26,30,31,40–42]. The difference across the study might possibly because of the difference in the level of medical care either in management strategies or antenatal care service quality. Additionally, the risk of newborn babies admission to NICU among women with HDP was higher as compared to babies from normotensive women; this was consistent with a study conducted in Nigeria and Tigray region [26,30]. The reason might be related to increased preterm birth, increased numbers of babies with low birth weight, and perinatal asphyxia as an adverse effect of hypertensive disorders of pregnancy.

The incidence of stillbirth among women with HDP was 11.2% with 95% CI of (7.63–15.68). This finding was consistent with studies report from Mizan Tepi (9.1%), Tigray region (10%), and Mettu (10.2%) [19,26,43]. Whereas the finding of this study was higher than the study conducted in Zimbabwe (5.4%) and Ghana (6.8%) [32,36], but lower than studies reported from Nekemte (22.1%) and Hawassa (23.5%) [31,44]. This difference could be due to the quality of antenatal care service and obstetrics care across health institutions and the study design used. Consequentially, the finding of this study was consistent with a higher relative risk for stillbirth observed among women with HDP than normotensive women, which was in line with a study conducted in Tigray region and Chine [26,45]. This might be related with the effect of maternal mal-perfusion and placental ischemia related to HDP.

This study also revealed that early neonatal death occurred among women with HDP was 11.3%. Moreover, the risk of early neonatal death was higher among women with HDP than normotensive women. Similarly, the incidence of perinatal mortality among women with HDP was 21.2% with 95% of CI: (16.42–26.73). This finding was higher than the study conducted in Ethiopia (Mettu 12.04%, and Tigray region 15%) [19,26], Ghana (10.6%), Nigeria (7.6%), and Madagascar (8.7%) [4,32,46]. The higher perinatal mortality discrepancy might also be attributed to the tertiary status of JMC which serves as the referral centre for the primary and secondary health facilities in the southwestern part of the country and small sample size recruited by others. This finding indicates a higher discrepancy from the sustainable development goal (SDG) target of neonatal mortality reduction to less than 12 per 1000 total births. This show that, it demands to strengthen maternal and newborn health care in order to achieve global and national SDG target plan by focusing prevention and treatment strategy of HDP and other predictors of perinatal mortality.

The strength of the present study is advantage large sample size and adjustment of potential confounders. The limitations of the study are that, variable that may have potential relationship with perinatal outcomes such as maternal nutritional status, smoking, indoor air pollution, maternal educational status, and wealth index were not included and also the data on the subtypes of HDP were not used for calculating the rate and the risk for each subtype.

## Conclusion

Higher incidence of adverse perinatal outcomes occurred among women with hypertensive disorders of pregnancy than normotensive women gave birth at Jimma Medical Center, southwest Ethiopia. HDP was associated with higher risk of preterm birth, low birth weight, low Apgar score, small for gestational age, stillbirth, admission to NICU and perinatal mortality.

Low number of antenatal care visit among women with hypertensive disorder of pregnancy than normotensive women was found in this study. This is associated with an increased risk of adverse perinatal outcomes. Therefore, provision of regular, high quality antenatal care and empowering women with accurate health information help to reduce adverse perinatal outcomes.

Hence, health care providers should strengthen prevention, early diagnosis and prompt management of HDP in order to improve better perinatal outcomes. Intervention towards HDP will signify to achieve global and national sustainable development goal target to reducing neonatal mortality in addition to other predictors of perinatal mortality.

Ethiopian Federal Minister of Health needs to set the criteria to identify pregnant women at high risk for HDP during antenatal care visit that may be used for secondary prevention. Other researchers need to include variables measured using prospective method.

## Supporting information

S1 File Minimum data set. (DTA)
